# Investigation of the endometrial receptivity status in experimental hypothyroid-induced female rats

**DOI:** 10.22038/IJBMS.2022.63143.13948

**Published:** 2022-09

**Authors:** Elif Erbaş, Semin Gedikli

**Affiliations:** 1 Department of Histology and Embryology, Faculty of Veterinary Medicine, Atatürk University, Erzurum, Turkey

**Keywords:** Endometrium, Hypothyroidism, Infertility, Propylthiouracil, Thyroxine

## Abstract

**Objective(s):**

This study aimed to investigate hypothyroidism’s effects on endometrial receptivity, creating an experimental hypothyroidism model in female rats.

**Materials and Methods:**

To induce hypothyroidism in rats of Hipotiroid-ER and Treatment-ER groups, 0.05% 6-propyl-2-thiouracil was freshly added to their drinking water for 8 weeks and then the endometrial receptivity model was applied and sacrificed on the fifth day. In the Treatment-ER group, after sc-administration of 0.8 µg/100 g L-thyroxine for 10 days, the endometrial receptivity model was applied to the rats and sacrificed on the fifth day.

**Results:**

In the histopathological evaluation, epithelial degeneration, vacuolization, enlargement of the uterine glands, and morphological disorders were observed in the endometrial layer of the Hypothyroid-ER group. However, these pathologies were significantly alleviated in the Treatment-ER group. Integrin β3, integrin αvβ3, LIF, and HOXA10 immune reaction intensities were high in the Control-ER and Treatment-ER groups, while in the Hypothyroid-ER group, integrin β3, integrin αvβ3, LIF, and HOXA10 immunoreactivity intensities were low. Also, while MUC1 immunoreactivity was high in the Hypothyroid-ER group, it was low in the other groups. In biochemical analysis, a significant increase in the TSH and progesterone levels and a significant decrease in the FT4, E2, FSH, and LH levels in the Hypothyroid-ER group compared with the Control-ER group were observed. Also, all hormone levels were significantly ameliorated in the rats of the Treatment-ER group compared with the Hypothyroid-ER group.

**Conclusion:**

The results obtained showed that hypothyroidism had a significant effect on endometrial receptivity—the histopathological and biochemical changes caused by hypothyroidism in the experimental rat model were ameliorated with L-thyroxine treatment.

## Introduction

Infertility is one of the most important clinical problems of our time. Every year, 10–15% of the population is diagnosed with infertility. Endocrinological diseases have an important place among the causes of female infertility (1, 2). Hypothyroidism is one of the most important of these endocrinological diseases and is quite prevalent (3). It is a result of insufficient levels of thyroid hormones T3 (triiodothyronine) and T4 (thyroxine) (4). The main reasons why endocrinological diseases negatively affect fertility are that ovulation, corpus luteum function, and endometrial receptivity, a very important factor in implantation, are decreased (5). Hypothyroidism, which is very common in women of reproductive age, adversely affects sexual maturation and the menstrual cycle, and causes delayed puberty, anovulation, amenorrhea or hypermenorrhea, and menstrual irregularity. In addition, it inhibits fertility and causes an increase in pathologies such as gynecological disorders, abortion, and fetal mortality (6-9). It has also been observed that the morphology of the uterine epithelium and the layer of the endometrium changed and degenerated. Whereas in order for quality receptivity and implantation to occur, a normal and healthy endometrium is essential (10). Certain factors, such as hormonal balance, healthy development of blastocyst, endometrial quality, and synchronization between endometrium and blastocyst, are effective for implantation to happen successfully. All of these very crucial events that occur between the blastocyst and the endometrium are called endometrial receptivity (11-12).

Pinopod formation and the synthesis of adhesive molecules are utterly important in this period when important interactions between the blastocyst and the endometrium are formed. The levels of integrin beta 3 (integrin β3) and integrin alpha v beta 3 (integrin avß3), which are expressed during this period, have been shown in many studies to be decisive factors for endometrial receptivity (13, 14). Leukemia inhibitory factor (LIF) and homeobox A10 (HOXA10) are among other molecules associated with this period (15, 16). It was also reported that mucin 1 (MUC1) expression in this period adversely affected endometrial receptivity (17). The studies conducted revealed that pinopod formation and the expression anomalies of adhesive molecules during the period of endometrial receptivity directly affected the success of pregnancy occurrence (13, 18).

In the present study, we aimed to examine whether hypothyroidism affected the endometrial receptivity of adult female rats by particularly evaluating the changes in the histology of the endometrium, and regulation of thyroid-stimulating hormone (TSH), free T4 (FT4), progesterone, estrogen (E2), follicle-stimulating hormone (FSH), and luteinizing hormone (LH) from different aspects.

## Materials and Methods

***Animal procedure***

The ethical approval of the study was obtained from Ataturk University Animal Experiments Local Ethics Committee (decision number: 13/224, date 12.27.2018). A total of 36 12-week-old Sprague-Dawley female rats weighing 200–250 g were procured from Ataturk University Experimental Research and Application Center. Before the experiments, the adaptation of the animals to the environment was ensured, and they were divided into groups. The rats were provided water and pellet food *ad libitum* throughout the study. The animals were housed in laboratory conditions of a 12-hour light/dark cycle at the temperature of 21–23 °C and relative humidity of 45–50% during the experiments.

***Animal groups***

In the experiment, healthy rats with normal estrous cycles were divided into three groups (n = 12). The first weight measurements of the animals were performed on the day the experiment started, and they were weighed once a week at regular intervals throughout the experiment.

***Experimental groups***

Control-Endometrial Receptivity (ER) group: The rats in this group were not subjected to any experimental treatment. At the end of the eighth week, the oestrus cycle was determined by the vaginal wash method.

Hypothyroid**-**Endometrial Receptivity (ER) group: The rats were given 0.05% PTU (6-propyl-2-thiouracil- Sigma P3755), which was freshly prepared daily, by mixing in their drinking water for eight weeks (7, 19). At the end of the eighth week, the oestrus cycle was determined by the vaginal wash method.

Treatment**-**Endometrial Receptivity (ER) group: The rats were given 0.05% PTU, which was freshly prepared daily, by mixing in their drinking water for eight weeks. After making sure that the hypothyroidism model was induced, the rats were subcutaneously administered with 0.8µg/100g L-thyroxine (Santa Cruz, sc-207813A) daily for 10 days (7). At the end of 66 days, the oestrus cycle of the animals was determined by the vaginal wash method.

***Hypothyroid animal model***

No drug treatment was administered to the Control-ER group. To induce a hypothyroid model in the rats in the Hypothyroid-ER and Treatment-ER groups, 0.05% PTU, which was freshly prepared daily, was given to the animals by mixing in the drinking water for eight weeks (7, 19). At the end of the fourth week, intracardiac blood samples were collected from two randomly selected animals from each group under anesthesia. Serum TSH (SunRed, Cat. No: 201-11-0181) and FT4 (SunRed, Cat. No: 201-11-0338) levels in the collected blood samples were analyzed with rat-specific Elisa kits and Elisa reader device (Bio Tek μ-Quant MQX200 Elisa reader/USA). The induction of hypothyroidism was biochemically determined, and the next steps of the experiment proceeded. 

***Estrus cycle***

**Figure 1:**
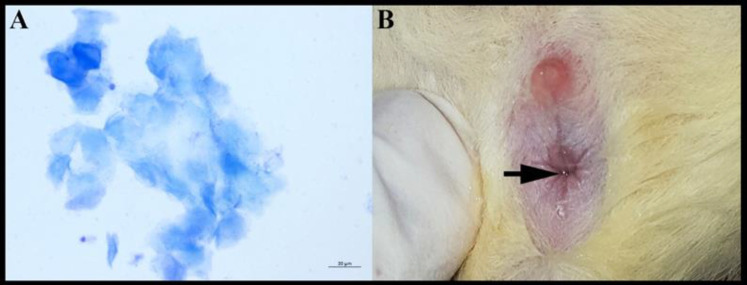
Illustration of the cornified cell (A) in the oestrus cycle and vaginal plaque (B) of rats. (Magnification:X400)

The oestrus cycle of rats in all groups was determined by the vagina wash method. The rats in the oestrus phase were detected by observing anucleated cornified cells specific to this period (Figure 1A) (20, 21). Animals determined to be in the oestrus phase were allowed to mate for one night in such a way that there were two female and one male rat (22). The next day, the males were separated from the female rats. The rats with a vaginal plug were regarded as pregnant (Figure 1B) (23), and that day was counted to be the first day of pregnancy. The fifth day was considered the endometrial receptivity phase by taking into account the studies in the literature, and the animals were sacrificed on that day (24). 

***Surgical procedures***

In order to terminate the experiment, the animals were anesthetized intraperitoneally with 100 mg/kg ketamine and 15 mg/kg xylazine, after collecting cardiac blood samples, they were decapitated and the uterine tissues were taken. The tissues taken for histopathological examination were directly placed into 10% formaldehyde solution. 

***Histological analysis***

The fixed uterine tissues were passed through graded alcohol and xylol series, embedded in paraffin blocks, and five micrometer thick serial sections were obtained from those blocks (Leica RM2125 RTS). The sections were used in histopathological and immunohistochemical staining. Crosman modified Mallory’s Triple Staining procedure was used for histopathological analysis.

***Immunohistochemical analysis***

The Streptavidin-Biotin Complex method was used for immunohistochemical staining. The antibodies used in immunohistochemical staining are Integrin β3 (Santa Cruz, 200 µg/ml, sc-365679), Integrin αvβ3 (Santa Cruz, 200 µg/ml, sc-7312), HOXA10 (Biorbyt, 100 µg/ml, orb-13476), MUC1 (Santa Cruz, 200 μg/ml, sc-53381), and LIF (Santa Cruz, 200 μg/ml, sc-515931). The primary antibodies used were diluted at the ratio of 1:50. All microscopic examinations and photography were conducted using a Zeiss AXIO Scope.A1 microscope equipped with a computer and a camera. A semi-quantitative scoring method was employed in the immunohistochemical evaluation of the study. According to the basis of this method, the scoring process was performed by taking account of the average staining intensity of at least 5 different areas from each uterine tissue (25). Immunopositive and immunonegative staining scores were determined based on the evaluations made as follows; very little or no staining: – (0%), low intensity: + (0–30%), moderate intensity: ++ (30–60%), and high intensity: +++ (60–100%) (26). 

***Biochemical analysis***

Blood samples taken into serum tubes were centrifuged at 3,000 rpm for 10 min at +4 °C. The serum samples obtained were stored in a deep freezer at -80 °C until biochemical analyses were performed. In the determination of serum TSH (SunRed, Cat. No: 201-11-0181), FT4 (SunRed, Cat. No: 201-11-0338), E2 (SunRed, Cat. No: 201-11-0175), FSH (SunRed, Cat. No: 201-11-0183), and LH (SunRed, Cat. No: 201-11-0180) levels, the rat specific ELISA commercial kits were employed in accordance with the procedure, and the measurements were made by a Biotek ELISA Reader device (Bio Tek μ-Quant MQX200 ELISA reader, USA).

***Statistical analysis***

A one-way analysis of variance (ANOVA) was performed using the SPSS 22.0 software package to determine the significance of the differences between the groups in the study. The Tukey test was used for the multiple comparisons of normally distributed data. The Wilcoxon signed ranks test was applied for pairwise comparisons within groups. A *P*-value less than 0.05 was considered significant.

## Results

***Animal body weights***

**Figure 2:**
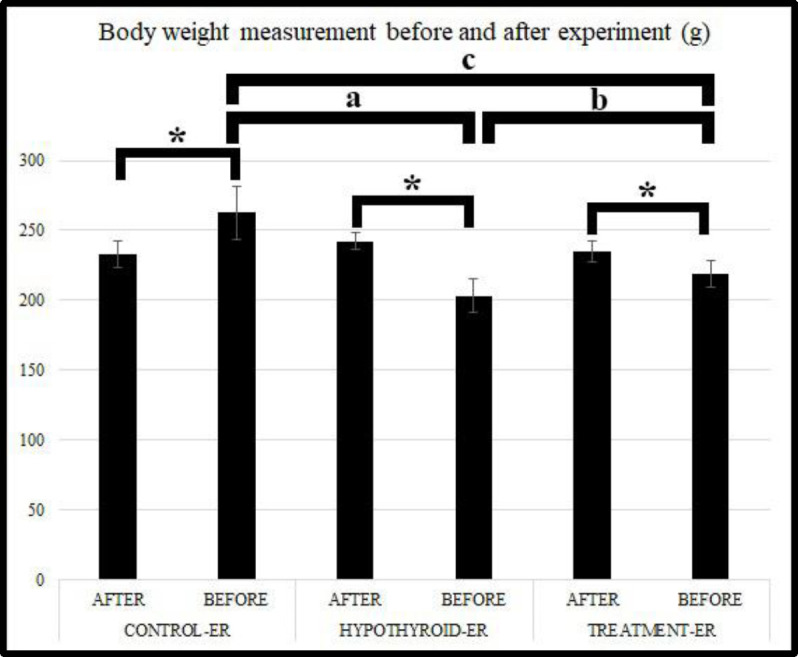
Body weight measurement before and after the experiment and comparisons between groups, (a ,b, c) different letters indicate the statistical differences between the groups. The asterisk (*) indicates the statistical differences within the groups

No difference was found between the groups in terms of body weight measurements made at the start of the experiment after the rats were divided into groups (*P*>0.05). In the comparison of body weights before and after the experiment, it was determined that there was a significant increase at the end of the experiment in the Control-ER group (*P*<0.05). In contrast, a significant decrease was observed in the Hypothyroid-ER and Treatment-ER groups (*P***<**0.05). However, the body weight was improved depending on the hypothyroidism treatment in the Treatment-ER group. The difference between the groups in body weight at the end of the experiment was found to be significant (*P*<0.05). Within and between comparisons of the groups in terms of body weight are presented in Figure 2. 

***Histopathological results in uterine tissue***

**Figure 3:**
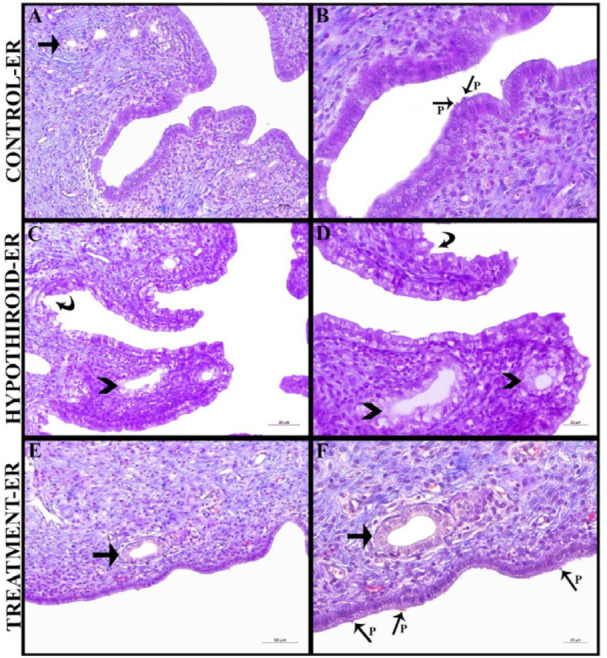
Illustration of histopathological changes of uterine tissues for all groups. Arrow (A, E, F): Normal uterine gland, P (B, F): Pinopod, Arrow Head (C, D): Degenerative uterine gland, Curved Arrow (C, D): Degenerative uterine lamina epithelialis (Magnification: A, C, E X200; B,D,F: X400)

In the histopathological examination of the uterine tissues belonging to the Control-ER group, plenty of pinopods were observed in the lamina epithelial layer of the endometrium and the structures of the uterine glands in the lamina propria layer appeared to be normal. According to the histopathological examination of the uterine tissues of the Hypothyroid-ER group, it was observed that vacuolization and degeneration were widespread in the lamina epithelial layer of the endometrium, while pinopod formations were not detected. Besides, degeneration of the uterine gland epithelia and enlargement of the glands were determined in this group. The histopathological examination of the uterine tissues of the Treatment-ER group, on the other hand, showed that the degeneration and vacuolization that occurred in the lamina epithelial layer of the endometrium were substantially reduced, and pinopod formation increased. It was observed that the uterine glands were normal in size, and epithelial degeneration decreased in this group. In general, a histopathological appearance of the Treatment-ER group was detected to be closer to the Control-ER group. Normal histological structure and histopathologies are shown in Figure 3.

***Immunohistochemical staining results of the uterine tissues***

**Figure 4:**
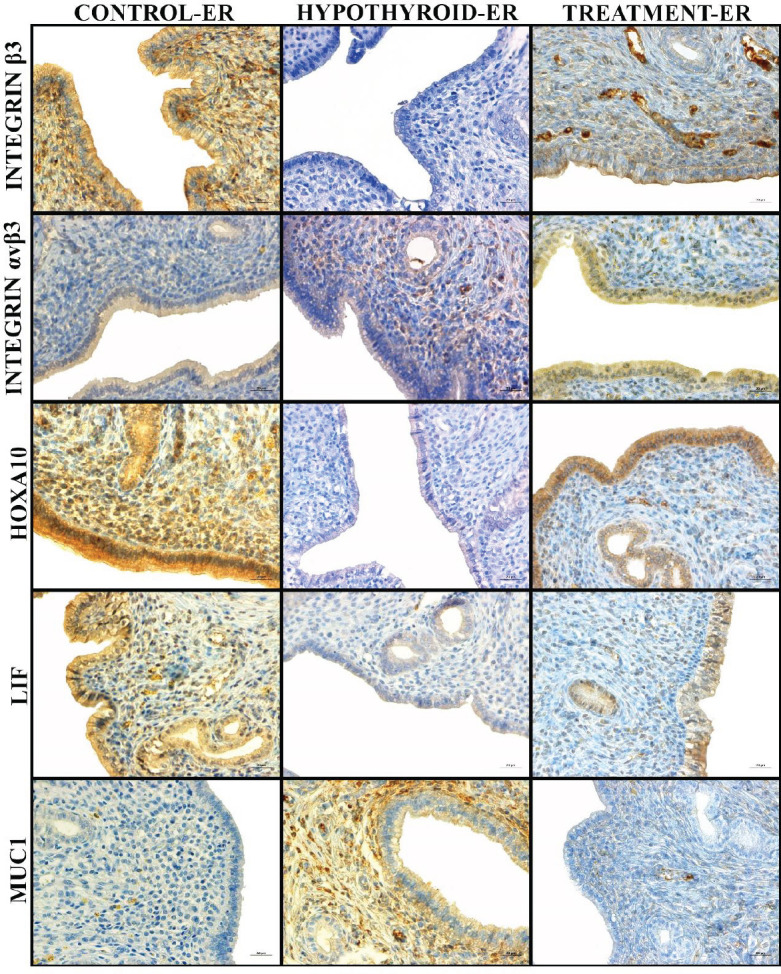
Immunohistochemical reactivities of integrin β3, integrin αvβ3, HOXA10, LIF, and mucin 1 (MUC1) antibodies for all groups. (Streptavidin-peroxidase staining)

Integrin β3, integrin αvβ3, HOXA10, LIF, and MUC1 antibodies were used for immunohistochemical staining. As a result of the immunohistochemical staining made by the integrin β3 antibody, the immunoreactivity intensity was high (+++) in the Control-ER group and moderate (++) in the Treatment-ER group, while no immunoreactivity (–) was observed in the Hypothyroid-ER group. The immunohistochemical staining of uterine tissues with the integrin αvβ3 antibody showed that the immunoreactivity intensity was low (+) in the Control-ER and Treatment-ER groups, on the other hand, there was no immunoreactivity (–) in the Hypothyroid-ER group. According to the results of the immunohistochemical staining made by the HOXA10 antibody, while a high level of immunoreactivity (+++) was observed in the Control-ER and Treatment-ER groups, no immunoreactivity (–) was detected in the Hypothyroid-ER group. Similarly, the results obtained from the immunohistochemical staining made by the LIF antibody revealed that while the immunoreactivity intensity was high (+++) in the Control-ER and Treatment-ER groups, there was no immunoreactivity (–) in the Hypothyroid-ER group. As a result of the immunohistochemical staining made by the MUC1 antibody, it was observed that the immunoreactivity intensity was high (+++) in the Hypothyroid-ER group, on the other hand, no immunoreactivity (–) was detected in the Control-ER and Treatment-ER groups. Immunohistochemical staining results of all groups are shown in Figure 4. Semi-quantitative evaluations made in terms of the intensity of immunoreactivity on the epithelial surface are presented in Table 1.

***Biochemical serum hormone concentrations***

**Figure 5:**
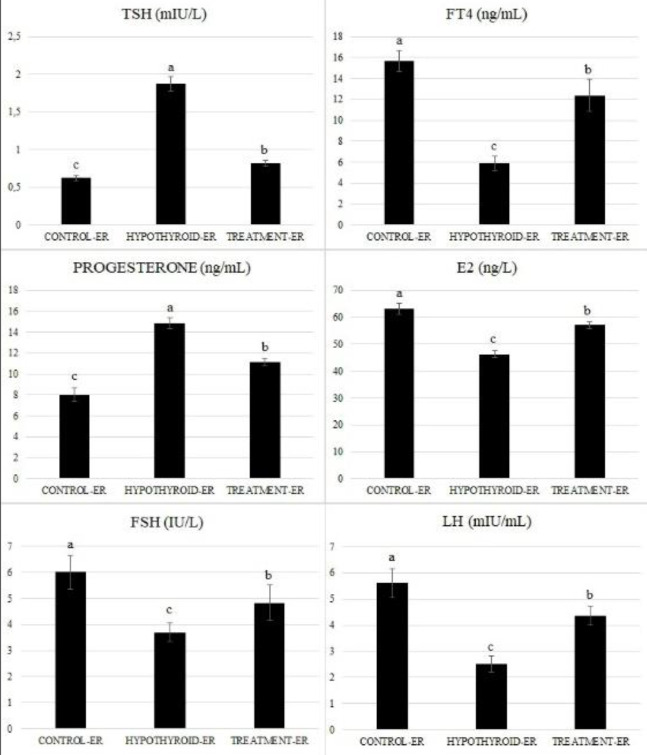
Biochemical serum TSH, FT4, Progesterone, E2, FSH, and LH concentrations for all groups. All values were given as mean and standard error (x̄±SEM). (abc) Different letters indicate the statistical differences between the groups

It was determined that serum TSH and progesterone levels were higher in the Hypothyroid-ER group than in the Control-ER and Treatment-ER groups. Those hormone levels were observed to decrease in the Treatment-ER group compared with the Hypothyroid-ER group, approaching the values obtained in the Control-ER group. (*P*<0.001). The TSH levels were 0.62, 1.87, and 0.82 mIU/L, and the progesterone levels were 8.03, 14.87, and 11.15 ng/ml in the Control-ER, Hypothyroid-ER, and Treatment-ER groups, respectively. The results of the statistical analysis revealed that all these differences were significant (*P*<0.001). A decrease in serum FT4, E2, FSH, and LH levels was detected in the Hypothyroid-ER group compared with all other groups. In contrast, these hormone levels were found to increase in the Treatment-ER group compared with the Hypothyroid-ER group (*P*<0.001). It was determined that the FT4 levels were 15.63, 5.91, and 12.37 ng/ml, the E2 levels were 63.21, 46.29, and 57.03 ng/L, the FSH levels were 6.01, 3.70, and 4.83 IU/L, and the LH levels were 5.61, 2.51, and 4.38 mIU/ml in the Control-ER, Hypothyroid-ER and Treatment-ER groups, respectively, and all these differences were found to be statistically significant (*P*<0.001). Biochemical serum hormone concentrations for all groups are shown in Figure 5.

## Discussion

While thyroid diseases affect many systems, such as the endocrine and reproductive systems, they also damage some organs in the body (27). Thyroid gland diseases can affect the fetus and newborn and the occurrence and course of pregnancy in women of reproductive age (28-30). In the present study, the effects of thyroid hormones on endometrial receptivity were investigated in hypothyroidism-induced rats.

In rats with hypothyroidism, myometrium and endometrium volumes, and thus uterine weights, are low (4,31). In the study conducted by Mangge *et al*. (32), weight loss was reported in the groups in which they induced hypothyroidism with PTU compared with the control group. Another study investigating the effect of subacute hypothyroidism on metabolism and growth detected a decrease in body weight (33). The present study also revealed weight loss in the treatment groups, and it was observed that there was an improvement in this bodyweight loss with hypothyroidism treatment.

Pinopods, the most important morphological biomarkers associated with endometrial receptivity, form between the 20th and 22nd day of the menstrual cycle and play an important role in implantation (34). Bagot *et al*. (35) showed that implantation success was associated with pinopods. Afami (36), on the other hand, reported that many molecules related to endometrial receptivity linked to pinopods, and those pinopods were helpful in implantation. In the histopathological examinations performed in the current study, plenty of pinopod formation was detected in the Control-ER group. In the Hypothyroid-ER group, vacuolization and degeneration were widely observed in the lamina epithelial layer of the endometrium, and enlargement and degeneration were also detected in the uterine glands. However, pinopod formations were not observed. In the Treatment-ER group, these pathologies were greatly reduced, and the presence of pinopod formations was discerned. In the study conducted on the rats, Kayhan (37) reported vacuolization and intense degeneration in the uterine epithelium in the hypothyroid groups, similar to our results.

Integrins play a role in early embryonic events, including implantation, fertilization, and formation of the first blastula (38). They are characterized on the embryonic surface with the endometrium during the implantation of species such as humans, rabbits, mice, sheep, and rats and are defined as markers of uterine receptivity (39). The integrin β3 and integrin αvβ3 molecules have been used as supportive findings in studies on endometrial receptivity and have been reported to have important roles in implantation failures (13, 40). In the present study, the integrin β3 and integrin αvβ3 molecules were used to show the endometrial receptivity status. The expression of the integrin β3 antibody was observed to be high in the Control-ER group and moderate in the Treatment-ER group. At the same time, there was no expression in the Hypothyroid-ER group. The integrin αvβ3 antibody results indicated that while immunopositivity was low in the Control-ER and Treatment-ER groups, there was immunonegative staining in the Hypothyroid-ER group. In the study conducted on mice with Hashimoto’s thyroiditis, Wu *et al*. (41) reported that the integrin β3 expression levels of sick mice decreased compared with the control group. In another study, the immunohistochemical staining results of the integrin avß3 in fertile and infertile women were compared, and it was found that the expression of integrin avß3 in infertile women was severely reduced (42). 

The HOXA10 molecule, which is highly important for implantation, is involved in the development of the uterus, endometrium, and endometrial stroma layer. The secretion of this molecule is regulated by hormones including progesterone and E2 (43). In the current study, it was found that the HOXA10 antibody was expressed in high levels in the Control-ER and Treatment-ER groups, on the other hand, it was not expressed in the Hypothyroid-ER group. It was reported in a study conducted on mice that the HOXA10 molecule is important for fertility (44). Similarly, the HOXA10 gene expression has been shown to decrease in the endometrial examination of polycystic ovarian cases (45). A study (46) revealed decreased HOXA10 expressions in endometrial samples of patients with uterine fibroids, unexplained infertility, and endometriosis in all patient groups. 

The leukemia inhibitory factor (LIF), a member of the interleukin 6 family, is one of the molecules required for embryo implantation (47, 48). The first evidence for LIF’s role in implantation was obtained by Steawart *et al*. (49) by observing that the embryos were not implanted in LIF-deficient female mice and that implantation occurred with LIF supplementation in the same mouse model. In the present study, we observed that the expression level of the LIF antibody was high in the Control-ER and Treatment-ER groups, on the other hand, the staining in the Hypothyroid-ER group was immunonegative. In studies conducted about unexplained infertility and recurrent implantation failures, a decrease in LIF expression or mutation of the LIF gene was detected in the treatment groups (50, 51). Researchers (52) found that women with weaker LIF immunoreactivity during the implantation window were less likely to get pregnant than women with higher LIF expression.

Mucins are in a glycoprotein structure and have a high molecular weight. Due to their high-water holding capacity, they are secreted on the surfaces of the lumen organs of the reproductive, digestive, and respiratory systems in order to moisten and lubricate the environment. Because of these properties, MUC1 is known as an anti-adhesive molecule and is associated with the endometrium (53). MUC1, which has a thick glycocalyx structure in the endometrium, is involved in adjusting the right place and time for the uterus in embryo implantation (17, 54). In the current study, intensive immunopositivity was observed in the Hypothyroid-ER group when the sample was subjected to the MUC1 antibody, while immunonegative staining was detected in the Control-ER and Treatment-ER groups. In a study in which epitopes on the endometrial epithelium were investigated by scanning electron microscopy, MUC1 was not found in pinopods (55). Another study showed a decrease in the MUC1 expression in the endometrium of women with recurrent pregnancy losses (56). Similarly, Bastu *et al*. (57) demonstrated that the MUC1 level was significantly lower in women with recurrent miscarriages than in healthy women. Many studies in the literature reported that low MUC1 expression was associated with impaired receptivity of the endometrium (58-60). 

When the biochemical results of the study were examined, it was determined that TSH and progesterone levels were significantly higher in the Hypothyroid-ER group, while FT4, E2, FSH, and LH levels were significantly lower. In the Treatment-ER group, on the other hand, TSH and progesterone levels were found to decrease significantly compared with the Hypothyroid-ER group, while FT4, E2, FSH, and LH levels significantly increased. A study (61) investigated the effects of hypothyroidism on hormones in mature female rats and reported that there was a significant increase in progesterone levels in the PTU group, the LH level was significantly lower in hypothyroid rats, the E2 and FSH levels in the thyroidectomy group tended to decrease compared with the control group, but there was no difference in terms of the FSH level between the PTU group and the control group. Likewise, Saleh (62) showed that the experimental hypothyroidism group had significantly lower T3 and T4 levels and significantly higher TSH levels compared with the control group. Another study indicated that the serum TSH level was 10 times higher in the hypothyroid group than in the control group, but there were no significant differences between the control group and the hypothyroid group in terms of serum LH, FSH, progesterone, and E2 levels (7). 

**Table 1:** Immunohistochemical semi-quantitative score values for Integrin β3, Integrin αvβ3, HOXA10, LIF, and MUC1 antibodies for all groups

Antibodies	Control-ER	Hypothyroid-ER	Treatment-ER
Integrin β3	+++	-	++
Integrin αvβ3	+	-	+
HOXA10	+++	-	+++
LIF	+++	-	+++
MUC1	-	+++	-

LIF: Leukemia inhibitory factor; MUC1: Mucin 1; ER: Endometrial Receptivity

## Conclusion

The results obtained in the present study showed that the disorders occurring in the experimental hypothyroidism model had a significant effect on the period of endometrial receptivity, and this condition can be returned to normal with administration of hypothyroidism treatment. At this point, there is a need for our findings to be supported by more detailed scientific studies.

## Authors’ Contributions

EE Provided the concept, histopathological findings, and immünohistochemical findings. SG Provided statical analysis, histopathological findings, and immünohistochemical findings.

## Funding Details

This work was supported by the Research Projects Coordination Unit of Atatürk University under Grant [TDK-2019-7261].

## Conflicts of Interest

The authors have declared that no conflicts of interest exist.
